# The Nature and Treatment of Cancer

**Published:** 1846-04

**Authors:** 


### The Nature ard Treatment of Cancer. By *W. H. Walshe*, M.D., Professor of Pathological Anatomy in University College, &c. &c. Octavo, pp. 590. London, Taylor and Walton. 1846.

The present elaborate and very valuable work is founded upon the article Cancer, that was written by the author for the Cyclopædia of Surgery, and published in that Dictionary about five or six years ago. A short time previously, the translation of Müller's important treatise on Morbid Growths had made its appearance, so that we were thus enabled to review the two works together, and give the English reader a full and fair account of the then-existing state of knowledge of the anatomy and developmental history of cancerous formations. Since that period, the subject has not again come under our particular notice; and we therefore gladly avail ourselves of the present opportunity of examining, at some length, the leading points in this most interesting, although (we grieve to say) still imperfectly-understood, theme of pathological investigation. It is one of the most painful circumstances to a man of considerate feelings, in the exercise of the medical profession, that there are certain diseases—and these too of by no means uncommon occurrence—which, from their very earliest detection, almost inevitably baffle every means for their cure, and, at a later period, often defy his best attempts at even temporary mitigation. Still he must not despond or despair; but, diligently making himself acquainted with all that has been done and may be doing in the way of scientific research, with the view of elucidating their nature, he must continue to cherish the hope that to some one, if not to him, may be reserved the proud satisfaction of at length discovering the means of arresting the growth, if not of preventing the early development, of a disease like Cancer. If such should ever be the case, we cannot believe that the discovery will ever be achieved by a chance thought or random guess; like that of Vaccination, it will rather be the glorious reward of patient observation, and of long and serious reflection.

With these remarks we invite the studious reader to follow us in our review of Dr. Walshe's work. It is divided into two parts, of nearly equal length. The first examines with great minuteness the entire subject of Cancer in general, embracing its anatomy, chemistry, physiology, pathology, and treatment; the second, is devoted to a description of the disease as seen in different parts and structures of the body, viz. the organs of digestion, respiration, circulation, and generation, of the lymphatic, nervous, and osseous systems, of the organs of sense, of the thyroid and thymus glands, &c. Our analysis will be limited almost exclusively to the first part of the volume.

First, let us consider the *Nosological position of Cancer*. To form any right ideas upon this subject, it will be necessary to premise a few remarks on what are usually called "Adventitious Products." By this term we mean all those substances which, although developed within the living body, yet form no part of its healthy structure, and the composition of which differs, more or less decidedly, from its natural constituents. These products Dr. Walse divides into two great classes, according as they consist of materials physiologically Inorganic, or physiologically Organic. In the first, he includes not only all saline matters in the form of crystals, pulverulent deposits, and concretions; but also certain proximate principles, which, though organic in a chemical point of view, are destitute of structure or organisation; as, for instance, oils, sugar, albumen. The second is made to comprehend all those that are said to be possessed of organisation. This class is a very large one; and, as it embraces very dissimilar objects, our author subdivides it into two families or subordinate classes, according as the new productions are, or are not, dependent "for existence on the immediate and direct access of nutritious matter from the blood of the parent organism." The former originate from a structureless fluid or *blastema*, and may therefore be called "Blastemal Productions;" the latter arise from a germ, and are therefore denominated "Germ-Formations or Parasites," being either of a vegetable or of an animal nature.

It is with the Blastemal Products that we have to do in considering the nosological position of Cancer; but, before we can yet determine this, we must make one or two further observations.

Blastemal Formations are essentially characterised by having a cell-structure. The nature and properties, however, of the cells, that are developed, differ very materially in different formations. 1. They may be utterly incapable of continued existence, or of generating any new cells previously to their own destruction. They therefore speedily become altered in their structure, and lose all traces of organisation, being either dissolved in the fluid matter around them, or disintegrated into an amorphous granular substance. Of this nature are Pus, Tubercle, and Colouring matter, as that of melanosis, &c. 2. The Cells, primarily developed in the blastema, may be non-vegetating, but persistent; in other words, they are not capable of generating others like themselves, but they possess the power of forming tissue more or less closely resembling some of the natural tissues of the body. The new formations, thus produced, are called "Pseudo-tissues;" thus we have pseudo-cellular membrane, pseudo-fibrous, pseudo-osseous, &c. tissues. 3. The primary cells may have a distinctly vegetative faculty, generating others like themselves previously to their own destruction, and these again in their turn being endowed with a similar reproducing power. To the morbid formations so produced, Dr. Walshe proposes to restrict the appellation of "Growths." He recognises but two kinds of these productions. In the one, the abnormal or diseased materials are merely accumulated in some part or other of the body; in the other, they are liable to become infiltrated amid the elementary molecules in the natural fissues of the affected part, causing an atrophous destruction of these molecules, and preventing the evolution of similar new ones; at the same time, they produce a similar disease in distant parts, they re-appear after destruction, and they give rise to a peculiar and characteristic cachexia, which invariably proves fatal. Thus, we have the subdivision of Growths into those that are Non-infiltrating and those that are Infiltrating. The first comprises the several genera of Fibrous Growths, Steatoma, Enchondroma, &c. &c. The second contains but one genus, and that genus is Cancer or Carcinoma.*

Under this generic appellation, Dr. Walshe describes not only the various forms of what have usually been called Scirrhus and open or ulcerated Cancer, but also that most formidable morbid growth which has been known at different times by the names of Spongoid inflammation and Tumour, Fungus Hæmatodes, Medullary Sarcoma, Encephaloid or Cerebriform Degeneration, Fungoid Disease, Cephaloma, Soft or Medul-

---

* The classification of Adventitious Products, proposed by our author, is far from being either simple or very satisfactory. Albumen and oily matters are considered to be inorganic; while tuberculous and even melanotic matter is said to be "possessed of organisation." The arrangement, adopted by Dr. Copland in the art. Disease in his great work, appears to us to be, although not unobjectionable, in some respects preferable to Dr. W's. He makes a three-fold division. The *first* embraces all adventitious products that are incapable of organisation or vitality; such as Pus, Tubercle, Fatty Matter, Glue-like or Colloid matter, and Melanosis. The *second* contains those that are susceptible of orgalary Cancer, &c.; and, lastly, that peculiar gelatiniform deposit or formation, which has been denominated Colloid matter or tissue, and which our author ranks as a distinct species of Carcinoma, in juxta-position with the former two.

Dr. Walshe begins with a description of—

*Encephaloid.*—This name was first applied by Laennec to a modification of cancerous growth, from its marked resemblance to the substance of the brain. Abernethy had previously called it, from the same circumstance, *medullary* sarcoma.

"Encephaloid tumors, like all other Growths, exhibit to the naked eye two kinds of component material,—a stromal or containing, and an intra-stromal or contained, element. The stromal substance, generally of denser structure than the other, divides the mass into minute loculi, lobules, and lobes. Its different divisions do not intersect, but describe curves in such manner as to circumscribe spaces, which, however various in size, have a general tendency to spherical form. Occasionally of fibrous consistence, even in their minuter divisions, the laminæ composing the walls of the loculi (in other words, the stromal* structure,) are generally of very delicate cellular texture, and have in some cases been compared by observers to a spider's web. The cerebriform appearance of the entire mass depends upon the contained, rather than the stromal, element." P. 13.

The consistence of the cerebriform matter may vary much in different portions of the same tumour. It may be as firm as fresh healthy adult brain; at other times, it is so soft and diffluent that it may be removed from its containing loculi by gentle pressure, or by letting a stream of water fall upon the morbid mass. The expressed substance occasionally preserves the shape of the loculi, and, oozing out like the matter from sebaceous follicles, looks like fragments of boiled rice or vermicelli.

Encephaloid structure is usually very vascular: in this respect, it presents a striking difference to the other two species of Cancerous growth. The vessels may often be seen to plunge from the stromal tissue into the contained cerebriform substance. In some successfully-injected specimens, the morbid mass has appeared to be almost exclusively formed of a vascular plexus. With respect to its form of deposition, or mode of connexion with the surrounding tissues, Encephaloid is either found as a distinct tumour, or it is infiltrated amid the elementary molecules of the part which form its *nidus*. The morbid mass is generally non-encysted; but sometimes it is inclosed in a pseudo-cyst, formed by the condensation of the adjacent cellular membrane, and the inner surface of this may have a serous appearance. Under such circumstances, the tumour has been known to

---

nisation; and this is subdivided into two groupes, according as the organisable products arise from sthenic inflammatory or hypertrophic action, and do not necessarily depend upon a depraved state of the constitution; or, as they originate in a constitutional vice, and may tend at the same time to vitiate the fluids and solids of the body more completely. In this class we find the various descriptions of tumours or morbid growths; the different species of Carcinoma belong to the last-named groupe or subdivision of these. The *third* comprehends those productions which are not only organised, but which possess an independent life: viz. all Entozoary Formations.

be enucleated with tolerable ease, as in some cases recorded by Dupuytren. Encephaloid occurs in the viscera in a peculiar nodular form; numerous tumours commonly co-existing in the same organ. The liver is particularly prone to this form of the disease, and may contain several hundred rounded masses, varying in size from a pea to a small egg. The lungs, also, are not unfrequently the seat of such nodules. Encephaloid growths acquire a larger size than almost any other sort of morbid formations, with the exception of compound cystoid and enchondromatous masses.

The following *varieties* of Encephaloid have been described; the physical characters of the morbid structure being found to vary a good deal in different cases.—1. Sometimes it is much firmer and closer than usual, resembling a good deal the boiled udder of a cow. This is the *mammary* or *mastoid* (so called by Abernethy) variety.—2. In other cases, it is hard, "of almost homogeneous appearance, of a pale yellowish colour, unctuous crisp-look, and possessing on the whole the visible characters of a sliced raw potatoe." This is the *solanoid* tumour of M. Recamier.—3. It resembles the milt of certain fishes. Dr. Monro 3tius, was the first to describe this variety under the name of the "*milt-like* tumour:" it is of very rare occurrence.—4. The *nephroid* cancer of M. Recamier, from some resemblance to the structure of the kidney.—5. The *fasciculate* cancer of Müller. "I am inclined," says our author, "to regard this as a distinct and important variety of encephaloid, but cannot, with Müller, consider it entitled to the rank of a species apart; for cells are sometimes to be discovered; and, moreover, filamentous or fibrillar structure, though to a much less amount, forms a constituent of other varieties of cancer."—6. When the brain-like character of the tumour is combined with an unusual degree of vascularity, it may be called *hæmatoid.*—7. The term *fungus hæmatodes* should be restricted to that variety of encephloid, "when interstitial hæmorrhage leads to sanguineous infiltration of the mass, or to irregular accumulations of blood through its substance; and when, especially after ulceration of the integuments, a rapid development of fungating growths takes place from its exposed surface."

In reference to the nomenclature, employed in designating these various forms of what is conceived to be essentially the same morbid formation, it would be well certainly (as Dr. Walsh observes) if some other specific appellation than that of encephaloid were adopted; and then the absurdity of speaking, for example, of the *nephroid* variety of *encephaloid* would be avoided. Dr. Walshe appears to prefer the use of the old term *soft cancer.*

*Scirrhus.*—By this term we are to understand "that species of cancerous growth which is distinguished by its hardness and toughness."* The practice of some writers, especially continental ones, applying the term to all tumours that are very hard, has introduced much and very serious confusion. Unless the morbid structure be at the same time of a malignant nature, and liable to terminate in intractable ulceration, it should not be called Scirrhous.

---

* Σκιρρος, Scirrhus, from σκιρος, a piece of marble.

Scirrhus, like encephaloid, invariably consists of a *stroma*, and of a diseased element contained within its meshes. When the mass is solid throughout, it often appears to be homogeneous, and then it is not easy to recognise the two structures of which it is composed. By allowing, however, the light to fall on the cut surface (which has usually a peculiar semi-transparent glossiness) of the tumor at different angles, or by submitting a slice of it to maceration, we shall generally be able to distinguish the stromal from the inter-stromal structure. The proportion of these two substances varies much in different cases, and, seemingly, according to the degree of development of the tumor examined. On this point, we shall allow our author to speak for himself.

"In a very early stage of its progress the stromal structure predominates, forming a firm, solid, pale, fibrous, or cartilaginiform-looking mass; at this period the deficiency of intra-stromal substance renders the stroma itself imperceptible unless upon close scrutiny. The similarity of scirrhus to fibro-cartilaginous texture under these circumstances has been recognised by numerous observers; M. Cruveilhier has even gone so far as to affirm that the only distinctive character (he speaks of those ascertainable with the unassisted eye) between some tumors in the bones, really scirrhous, and others merely fibrous, consists in the presence of cancerous juice (removable by pressure) in the former. The diagnostic importance of this fluid is unquestionable; but I believe that the two kinds of growth may also be distinguished by the rectilinear arrangement of the stroma of scirrhus, and the curvilinear of that of fibrous tumors.

"At a more advanced period a softer matter accumulates in the meshes of the stroma, and the locular character grows more obvious." P. 19.

The colour of the cut surface of a scirrhous tumour is a flinty blueish-white, when the mass is very firm; a lilac-yellow, pale dirty fawn, or greyish tint prevails, when there is much inter-stromal matter. The hardness is sometimes very great: hence the common appellation of "stone-cancer." This character is always much more remarkable when the tumour is *in situ*, than after removal.

By pressure, a fluid may be forced to exsude from the substance of scirrhus. When the tumour is very hard, this fluid is thin and watery; but, when less consistent, it is thicker, more opaque, inclining to a creamy appearance, and may be tinged with blood. The dull-white opaque filaments, that are often observed to be irregularly scattered through the substance of a scirrhous growth, are nothing more than lymphatic vessels, or in the mamma, lactiferous tubes. Small masses of Colloid are often found within the substance of a scirrhous mass. Scirrhus formation is unquestionably vascular, notwithstanding the assertion to the contrary of Scarpa, Travers, Lobstein and others; its blood-vessels, however, are very singularly and unequally distributed. "Patches of some size, which exhibit no trace of blood-vessel, may be seen in the vicinity of others sufficiently vascular; but the physiological inference, deducible from this fact, is not of the importance it would have possessed previously to the discovery of the mode of nutrition of extra-vascular structures."

Like encephaloid, Scirrhus is either developed as a distinct tumour, or it is infiltrated in the substance of some organ. Some writers have contended that scirrhus never occurs as a primary and idiopathic disease, except in some description or another of secretory tissue. This opinion has been amply shewn to be quite fallacious. The bones, the voluntary muscles, the heart, the lungs, the penis, &c., may be primarily affected. Scirrhus is rarely, if ever, invested in a true cyst; although Dupuytren, Recamier and others describe the occasional existence of such an envelope.—The infiltrated disease is especially common in the uterus. When this form attacks the bones, the osseous structure gradually becomes softened, while the soft tissues become indurated; thus affording, as Dr. Walshe remarks, a striking illustration of the assimilative power of the morbid growth; for, in each instance, it alters the consistence of the tissue affected to its own standard.

Scirrhous tumours are rarely, if ever, very large; they seldom exceed an orange in size. This character serves, in part, to distinguish them from fibrous growths.

The varieties of Scirrhus, that have been described, are—1, the *pancreatic*, from its alleged resemblance to the tissue of the pancreas; 2, the *chondroid*, from its dense and crisp structure like that of cartilage; 3, the *lardaceous*, from being not unlike to the boiled rind of bacon; 4, the *napiform*, from exhibiting on its divided surface the appearance of bands, the arrangement of which is such as to produce some similarity to a cut turnip; 5, the *apinoid*,* from its striking resemblance to the cut surface of an unripe pear, in consequence of "the dissemination of comparatively opaque, almost buff-coloured, spots through a more translucent ground of very pale yellowish lilac tint." This variety unquestionably belongs to the *carcinoma reticulare* of Müller. 6, Scirrhous structure is occasionally, though rarely, highly vascular; this may be called the *hæmatoid* variety of the disease.

*Colloid.*—This term was applied by Laennec to adventitious products having a gelatiniform appearance (κολλη, glue, and ειδος, form). There is one form of Cancer, in which the inter-stromal, or rather intra-locular, substance presents this feature; and it is to this malignant formation that Dr. Walshe wishes to restrict the term. Laennec and other writers since his time have, in Dr. W.'s opinion, erred seriously in associating with true colloid structure all adventitious matters which have a jelly-like aspect. The "gum-cancer" of Hodgkin, and the "carcinoma alveolare" of Müller, belong to this species of cancerous disease. Dr. Carswell regards Colloid as a mere variety of Scirrhus; and doubtless many will agree with him. The following excellent description will enable our readers to distinguish the true Colloid structure (as our author understands this term) at once.

"The section of a colloid growth presents an appearance which, once seen, can scarcely be forgotten. The surface is divided into a vast number of distinct loculi, regularly arranged, of an oval or rounded shape, varying in size from that of a grain of sand to the largest pea. The septa composing the walls of these loculi possess distinctly fibrous characters; their thickness is pretty uniform throughout; occasionally, however, they are broader in some situations than in others; in this case, the thicker septa may generally be found to give off productions forming the walls of secondary loculi, and these again others, constituting a tertiary order. The loculi sometimes form shut sacs, in other instances communicate with the circumjacent cells; it is not very unusual to observe some of these loculi, in which the walls seem to have collapsed and almost coalesced from the removal of the contained matter. In point of consistence a colloid mass, of which the loculi are perfect, usually resembles firm cheese, but may be much harder. The general colour of the divided surface is greenish-yellow,—the tint being mainly dependent on that of the contained matter, which is, besides, semi-transparent, tenacious, and clammy, and resembles in respect of density, as in other physical properties, soft jelly. It is not easily expressible from the containing loculi, but may be picked out with the point of a scalpel, or removed by maceration." P. 26.

Colloid is indistinctly, if at all, vascular. It is met with either as a distinct tumour, or infiltrating the tissue that is the seat of the morbid formation. The latter form occurs most frequently in the stomach, the walls of which sometimes become so exceedingly thickened in consequence, that the organ does not collapse when removed from the abdomen: it is met with likewise in the intestines and omentum. Dr. Walshe has never seen disseminated nodules of colloid in any of the parenchymatous organs: small spots however of infiltration in the omenta sometimes exhibit this appearance. In a subsequent part of his work, our author alludes more particularly to the common seat of colloid cancer. His words are :—

"The organs and parts in which I have myself actually seen this species of cancer are the stomach and adjacent glands; the small and large intestine; the omentum and the female breast.—An organ in which I have not seen it, but in which there can be no doubt of its having occurred, from the accuracy of the description, is the spinal marrow.—Organs and structures in which its occurrence is doubtful, are the lung and tendon.—Organs and parts in which other products have obviously been mistaken for colloid cancer, are the bones, the ovaries, the uterus, and the extremities.

"Cruveilhier, in associating colloid cancer with osteo-sarcoma and spina-ventosa, has evidently been deceived by some of its physical similarities to enchondroma. When he speaks of this cancer occurring in the ovary, his observations are to be understood really to refer to compound cystoid formations; and lastly his cases of pultaceous colloid cancer of the uterus appear to me examples of encephaloid infiltration, in which the locular character is marked with unusual clearness.

"Again, I have carefully examined some specimens of the gelatinous-looking masses, which occur with some frequency at the upper part of the arm, and give the part the exact outline of a shoulder of mutton. In no single one of these cases was the structure that of colloid cancer; but either of enchondroma or compound cystoid formations, associated sometimes with fatty matter in considerable quantities." P. 100.

Colloid growths sometimes acquire a large bulk: they have been found in the omentum as large as a cocoa-nut. Two-thirds and more of the anterior and posterior surfaces of the stomach have been seen infiltrated with the diseased matter.

Cruveilhier has described a variety of colloid which he calls "alveolar cancer *à matiere perlée*," from the white, pearl-like substance of the intra-locular matter. Müller, however, regards this substance "as a granular deposit of the peculiar variety of cholesteric fat, to which he has given the name of *cholesteotoma*."

The French pathologist has described a second variety, in which the inter-stromal matter is opaque, of a yellowish hue and tallow-like aspect, with granular fracture and feel, and the chemical composition of casein. This is the *areolar pultaceous Cancer* of Müller. Dr. Walshe suspects that this variety is rather encephaloid than colloid in its characters.

CHEMISTRY OF CANCER.

Cancerous substance, in a chemical point of view, is regarded by Müller and other enquirers as a protein-formation: "it belongs essentially to the *albuminous* variety, although both *fibrine* and casein enter (in general to a comparatively slight amount) into its composition." Several chemists have maintained that *gelatin* enters more or less abundantly into the composition of cancerous formation; but others have not detected a particle of this constituent in it.

Müller arranges all adventitious growths, in respect of their chemical composition, in three classes; viz. the albuminous, the gelatinous, and the fatty. He places all cancerous formations among the first set; and ascribes, with much plausibility, the occasional existence of gelatin in them to the presence of some cellular membrane along with the morbid structure. Even in the jelly-like matter of Colloid, when it is carefully freed from membranous structure, gelatin is rarely found to be present; it seems to be very generally nothing but a peculiar form of albumen.

Alcohol acts very differently upon the different species of cancer. The gelatiniform matter of Colloid retains its transparency in it, while scirrhous and especially encephaloid substance are rendered perfectly opaque.

The ultimate microscopical cells of cancer are insoluble in cold and boiling water, and are not seriously affected by acetic acid—characters distinguishing them from the red and white corpuscles of the blood, and also from those of purulent matter.

We need scarcely say that practical men should be very cautious in attaching much consequence to the chemical composition of morbid organized productions.

Does not Dr. Walshe venture very unnecessarily into the region of mere speculation, when he suggests what *may be* the chemical qualities of cancer-blastema? literally we know nothing on the subject; all is fond fancy and guesswork.

PHYSIOLOGY OF CANCER.

*Origin of the disease.*—It is quite unnecessary to do more than simply notice the various doctrines, that have been proposed by different theorists on this very obscure subject. Cancer has been attributed to the presence of parasitic animalcules, of a peculiar species of hydatid, of an animal fungus possessed of independent vitality, and so forth; but the conclusive refutation of these hypotheses, in our author's opinion, is to be found in the simple question, who has ever seen or demonstrated the presence of these formations?

Broussais and his followers have absurdly maintained that cancer is the result of mere inflammatory action. The folly of this notion is admirably exposed by our author in these remarks:

"The sophistry of the system of argument by which the doctrine of irritation was upheld is notorious: but the facts that simple induration is a state distinguished by its inactivity, while scirrhus possesses in itself an active principle of increase and growth; that carcinoma assimilates to its own nature the tissues it attacks, while inflammatory induration is modified by the particular structure affected; that the substance of cancer may from the first be almost pulpy, instead of necessarily possessing any marked degree of hardness; furnish a better refutation of it than any critical exposure of its fallacies. The former important result of clinical observation is confirmed, while it is explained, by microscopical investigation. On the one hand, simple induration-matter (as Müller was the first to state) does not contain cells provided with the germs of a new generation of similar structures; while, on the other hand, an inherent faculty of growth is produced and maintained in cancer by the formation of cells, which do contain such embryos of secondary generations." P. 38.

The ingenious doctrine of Dr. Hodgkin that cancer is produced by, and essentially consists in, the presence of simple or compound encysted structure, is generally admitted in the present day to be untenable. That carcinomatous matter is occasionally associated with the multilocular cysts of the ovary and mamma, may be admitted without recognising any necessary connection between the production of the two formations.*

We come now to notice, and that very briefly, the opinions of M. Cruveilhier and Dr. Carswell.

The former of these distinguished pathologists maintains that "the formation of cancer, like all nutritive phenomena, healthy and morbid, takes place in the venous capillary system; that from the minute veins the morbid products are poured into the cellular membrane, either by exhalation or through lacerated openings." The latter, also, has advocated the doctrine that the cancerous matter exists ready-formed in the blood, and that it becomes separated therefrom after the manner of nutrition or secretion, either in the molecular structure, or on the free surface, of organs. The act of cancerous deposition is thus regarded rather as one of depraved nutrition of the part affected, than of any actual transformation or degeneration of existing tissues. But, while he believes that "a modification of the blood constitutes the primary, if not the essential, condition of the formation of the disease," he admits, in the very next sentence, that "we can offer no explanation of the nature of the change effected in the blood, nor, consequently, of the nature of carcinoma."

The only argument of any weight in favour of the primary production of the *materies morbi* in the blood, is the circumstance that carcinomatous matter has been found in the interior of the veins, not only of the affected part, but of organs at a distance from it. Dr. Walshe is not, however, satisfied of its force; and he thinks that it is much more probable that the morbid matter, in such instances, had found its way into the cavity of the veins by absorption from some part or organ in which cancer was already developed, than it had been primarily generated within the vessels themselves. He suggests, also, that the exhalation of ready-formed cancerous

* The cysts, which are not unfrequently found in the interior of cancerous growths, are suspected by our author to be exactly similar, in their mode of origin, to apoplectic cysts in the brain: a coagulum of blood has been effused, its solid constituents have become gradually absorbed, and a cystiform bag remains.

matter from the very minute capillaries is a physical impossibility (in consequence of the size of the diseased globules or nucleated cells), unless on the gratuitous hypothesis that the coats of these tubes have undergone some change.

Having disposed of the doctrines of others, our author proceeds to expound his own views as to the development of cancer. He supposes that the first act consists in the exudation of what he calls a *blastemal or germinal fluid*, which is in short nothing more than the liquor sanguinis, (supposed to be) somehow or other modified in its vital properties.* In what this modification exists, Dr. W. is, as a matter of course, quite unprepared to say. He is, however, of opinion that, most probably, it takes place within the vessels, *while the blastema is still mixing with the general circulating fluid.* If such be supposed to be the case, we can perceive little or

---

* Is not this very nearly the doctrine of Abernethy in different language? He referred all adventitious formations to the coagulable part of the blood as their origin: this he supposed to be effused into the cellular or parenchymatous tissue, or on the surface, of organs; then to become organised, and to derive the materials of its subsequent growth from the vascular system of the surrounding parts. Dr. Copland's views are nearly the same, as developed in his excellent remarks on *Morbid secretions associated with morbid nutrition, or secretions susceptible of organisation.* The following passage may be appositely introduced here:—

"The class of productions, in addition to a small proportion of the constituents of unorganised secretions, contain a large quantity of fibrine. M. Andral supposes that a small portion of this substance, either coagulated in the blood-vessels, or extravasated into or upon the tissues, is the original source whence the organised productions are formed; the fibrinous deposit presenting the appearance of a whitish or reddish mass, of variable consistence, and having a tendency to become organised, although at first possessing neither organisation or vitality. But I believe that all fibrinous exudations have a certain degree of derived vitality, disposing them to organisation, particularly when they continue in contact with the part that produced them. This pathologist considers, that a portion of fibrine may, when coagulated, indicate its vitality without presenting any blood-vessels or any determinate texture; in which state it may be compared to a zoophyte, which performs a certain grade of vital function, although destitute of a circulating system; and that the fibrinous mass, when impregnated with life, becomes the seat of various organic actions; has a tendency to assume the form of some one of the simple or compound animal textures; performs the functions of secretion, and exhibits the same morbid phenomena, when irritated, as the natural tissues do under similar circumstances. He further supposes that several tumours, the origin of which has hitherto been mistaken, may be traced to the solidification of fibrine in the blood-vessels of the part; and adduces cases, from the minute dissection of which, he infers, that many of the adventitious productions usually called *cancerous, sarcomatous, encephaloid,* and *medullary,* are entirely formed in this manner; the minute vessels—arterial, capillary, and venous—being filled with solid fibrine deprived of its colouring matter. It appears, however much better established, that the *latter* especially of these productions are formed chiefly of coagulated or altered fibrine, thrown out of the blood-vessels owing to their perverted action, and either collected in masses, into which blood-vessels are produced, or infiltrated into the tissue of the part, the vascularity of which is increased along with the alterations that supervene in the adventitious formation and its containing structure." Vol. I. p. 590-1.

no difference between the hypothesis of Dr. Walshe and that of Dr. Carswell. As to saying that Dr. C. holds the opinion "that the material elements or solid constituents of tumours exist primarily in the blood, and in fact circulate in suspension in that fluid, before they are aggregated into masses," the allegation seems to us to be scarcely warranted by any part of his writings. Surely Dr. Walshe is too niggard of his praise to his distinguished predecessor in the chair of Pathological Anatomy at University College; there is not one expression of cordial admiration of his merit, throughout the present volume.

On the whole, it must be frankly confessed that, after a great deal of ingenious speculation and of very learned language has been expended upon the subject, we really know little or nothing as to the *origin* or proximate cause of Cancer. The *structure and mode of growth* of the morbid formation, when this is once fairly developed, is more within the field of our observation.

"The ultimate solid constituents," says our author, "of carcinoma are granules; nucleated cells of spherical, elliptical, irregular, or caudate shape; free nuclei; and fibrillar substance: with these is associated in variable quantities a semi-transparent fluid,—blastema obviously apt for the generation of new solids. Capillary vessels are always discoverable. With these essential elements fat-globules are, from the constancy of their occurrence, almost entitled to take place. Compound-granule corpuscles, melanic matter in the form of cells and free-granules, saline matters, and certain other substances occur as accidental and contingent elements."* P.55.

For a minute description of each of these constituents, we must refer our readers to the original: all that we shall do is very briefly to notice the *vascular supplies* of Cancerous growths. The blood-vessels are found to be of two kinds; 1, ramifications from the vessels of the surrounding tissues; and 2, newly-formed and independent vessels, which originate *de novo* in the morbid structure, and whose contents are found to exhibit (at least for a time) an oscillatory movement quite unconnected with the general circulation: the former are called by Dr. Carswell the *collateral*, the latter the *proper*, vessels of the tumour. These two sets of vessels gradually inosculate with each other; but, as new ones are continually forming, there are always some that are isolated and independent. The softer the tumour, the more numerous in general these are. The following appearances in the gradual development of these *proper vessels* may often be traced with the naked eye:

---

* In a subsequent part of his work, Dr. Walshe, when alluding to the meaning of the epithet "malignant," as used by surgical writers, distinctly cautions the reader against attaching undue importance to the *microscopic characters* of cancerous formations. "Müller," says he, "admits that there are characters by which 'malignant' may be distinguished from 'benignant' growths, but they are only appreciable by the naked eye or with a lens, and lost under the microscope,—an opinion in which I perfectly coincide. In fact, *in the present state of knowledge,* chemical and microscopical examination would (while they indubitably in many cases aid greatly in distinguishing growths of different properties and tendencies) often lead us, on the other hand, into the error of confounding growths perfectly dissimilar in nature and effects." P. 188.

"In one part of a tumor may be seen some minute unconnected points of blood; at another, appear a number of such dots united in linear juxta-position, so as to form a streak of blood uninclosed in any distinct vessel; elsewhere, a vascular investment is found for a similar streak; further on, a similar piece of delicate tube divides at each extremity into a number of tapering ramifications, assuming a stellate or tufted arrangement." P. 60.

It has been disputed whether these new vessels are veins or arteries. Cruveilhier calls them veins; and, certainly, they have more of a venous than of an arterial aspect. They are frequently varicose; their walls are always very thin: hence their extreme tendency to become ruptured, and give rise to alarming hæmorrhage. The rapidity of the growth of a carcinomatous swelling is usually proportionate to the degree of its vascularity. Much also, in this respect, depends upon the degree of compression to which it is subjected. The rapidity of the growth of an Encephaloid, which perhaps may have been nearly stationary for years, after it has burst the skin, is too well known to every surgeon. Internal growths also sometimes increase with extraordinary speed. Andral has related a case of encephaloid tumour of the omentum, which so rapidly extended downwards as *daily* to acquire an increase in size, that was perceptible both by the eye and the hand: the patient was carried off within five weeks from the first appearance of the swelling.

*Process of Decay.*—All cancerous formations exhibit a tendency, after a varying lapse of time, to a process of inherent degeneration and decay, preparatory to their more or less complete elimination from the body. The morbid mass becomes softer and softer, until it is, either generally or partially, reduced into a pulpy or still thinner matter. Softened encephaloid has a creamy or milky look (hence the term "galactomyces," applied to it by Ritgen); its consistence varying between that of soft cheese and of thin pus.—The softening of scirrhus is usually much more gradual, and takes place also more irregularly in different parts of the morbid mass: on compressing it, either a thin turbid brownish-coloured fluid, or a thick, opaque and creamy one, or a cheesy-looking matter, not unlike that of the sebaceous follicles, may be squeezed out from its substance.—The jelly-like contents of Colloid undergo but little change.

The softening process of a cancerous growth has been very differently explained by different writers. Drs. Carswell and Hodgkin regard it as the result of mortification or gangrene, arising from obstruction of the circulation through the tumour, either from its veins becoming filled up with cancerous matter or from the compression of the adjoining tissues upon its substance. Other writers have ascribed the change in question to inflammatory action having been set up in the morbid substance. That such is the case in some instances is evident from the admixture that is occasionally observed of pus-corpuscles with broken-down fibres and cancer-cells. In others, however, we can observe no evidences of any inflammatory process having ever taken place. The softening may perhaps be most reasonably attributed to an internal spontaneous degeneration, similar in nature to that described by M. Gulliver as occurring in fibrine when retained in the interior of blood-vessels (*vide* Med.-Chir. Review, No. 63, January, 1840).

*Elimination.*—While the process of softening is going on in the substance of a carcinomatous tumour, the surrounding tissues usually become the seat of inflammatory and suppurative action. In a few cases, the investing cellular membrane sloughs away entirely, and the morbid growth has been known to be thus fairly detached and expelled from its *nidus.* When seated under the skin, the superjacent integuments become gradually thinner and thinner, either from atrophy or ulceration, until at length they give way; then we have what is called an "open Cancer." Through the outward opening thus made, the cancerous matter is expelled, either as an ichorous discharge, or in masses: this latter mode of separation occurs chiefly in encephaloid. The progress of this species of cancer is, it is well known, much more rapid after the skin has broken than that of scirrhus.

Cancerous ichor or Sanies is found to exhibit, under the microscope, "the elements of cancer in a state of more or less complete disintegration, —the cells being sometimes reduced to fragments, sometimes retaining their outline perfectly. With these elements are associated oil-globules, crystals, epithelium-scales, pus-corpuscles, blood-corpuscles, and certain elements of the structures (or of the secretions of these), amid which the ulcerating cancer is seated. Cholesteatomatous matter, as has long been known, occasionally forms on the surface of ulcers and sinuses of various kinds; and Müller has found in one instance the peculiar non-nucleated polyedral cells of this variety of fatty product, forming a thick layer of substance like tallow, on the surface of a cancerous ulcer in the mamma. When such a formation exists on the surface, its elements will of course be occasionally found in the discharge."

Infusory animalcules are not unfrequently discoverable in cancerous ichor. The occasional presence of these has actually led some hasty writers to attribute the production of the morbid growth itself to animalcular development.

Occasionally, though very rarely, cancerous ulcers have been known to assume a healthy appearance after a time, and even to become cicatrized over a greater or less extent of their surface. But in every such instance the temporary amendment has proved to be but a delusion; the lull before the outbreak of the storm.

### PROPAGATION OR DISSEMINATION OF CANCER.

In some cases the morbid growth appears to be confined, from first to last, to the single organ in which it arose; no other organ or part of the body exhibiting upon dissection any traces of infection. The proportion of such cases to the total number, Dr. Walshe declares to be "far from being contemptibly small."

The spreading of the disease takes place in a variety of ways. The parts, immediately surrounding the original spot of the mischief, may become infiltrated with the morbid matter. On the subject of *cancerous infiltration,* Dr. Walshe remarks:—

"This is the characteristic and most essential of the changes under consideration. Extension of a cancerous growth to contiguous textures by the process of infiltration occurs in the case of the three species, but not with the same facility and frequency. *Encephaloid* has been by some considered more prone to push aside, than to infiltrate, the adjacent parts: Abernethy considered this a fact of sufficiently constant occurrence and importance to warrant a complete nosological separation of scirrhus and 'medullary sarcoma.' But it is far from unusual for encephaloid of the cellular membrane of the limbs to spread to the adjoining tissues. Cerebriform growths originating under the peritonæum spread to the bones of the pelvis, and even of the thigh. This progressive destruction even of the hardest tissues was, indeed, by Ruysch esteemed so important, that he named the disease 'ossivorous tumor.' I have myself observed not a few cases, in which the infiltration of the muscular structure, consequent on the presence of a tumor in the cellular tissue, was so perfect, that the former seemed formed of fasciculi of encephaloid fibres. Such facts are so common, that it is needless to adduce further illustration. Dr. Hodgkin goes so far as to affirm that encephaloid is more prone than scirrhus to affect the surrounding tissues by infiltration. In this extreme opinion he is, according to my experience, certainly unsupported by facts. In the female breast, for instance, while infiltration of the subjacent muscular and other structures is an invariable occurrence, if the disease be scirrhus, I have seen not a very few cases of advanced encephaloid, where the morbid mass had not spread to those textures.

"The extension of *scirrhus* to contiguous textures is a very constant phenomenon; as it spreads, the scirrhous growth loses its circumscribed, rounded, moveable character.—No less frequent is the propagation of *colloid* in this manner." P. 89.

That certain extra-vascular tissues are acted upon, and eventually destroyed by, the progressive advance of cancerous disease is well known to surgeons; but the mode, in which this destruction takes place, has not yet been fairly made out.

"Articular cartilages may be adduced as exemplifying the circumstance; and it appears to me perfectly consonant with reason, and supported by the very strongest and most direct analogy, to suppose that this extra-vascular tissue imbibes blastema thrown out by the vessels of juxta-posed vascular structure, and that this blastema subsequently goes through the same series of changes as if infiltrating a vascularized tissue. It is a thoroughly established and familiar fact, that though the cornea ranks among structures deficient in blood-vessels, and is therefore incapable of undergoing the earliest or congestive stage of inflammation, still it becomes affected with the second stage of that process, by imbibing exudation-matter effused in its vicinity by the vessels of circumjacent vascular structures. This truth, which is so conveniently demonstrable on account of the natural transparence and acquired opacity of the cornea, is more difficult to prove, but, I am persuaded, not less real, in the case of cancer of the articular cartilages." P. 54.

The mere juxta-position or close proximity to parts affected with cancer may induce contamination in certain tissues, although there is no continuity of substance between them. Thus, when the ovary, mesentery, or liver is the seat of cancer, the parts in contact with these organs, as the intestines and abdominal parietes, are often found to have become infected. The transmission of the disease to parts, at a distance from the seat of the primary evil, may be effected through the medium either of the lymphatics, or of the veins. It used to be the custom of medical writers to refer the more frequent development of secondary cancer, as of secondary abscesses, in certain organs than in others, to a peculiar *sympathy* existing between the parts affected. But this is a mere fancy, utterly unsupported, or rather directly contradicted, by accurate observation. For example, it is now an admitted fact, that the mamma is very rarely involved when the uterus is primarily affected; and, on the other hand, it would seem, from indubitable evidence, that the uterus ordinarily remains perfectly free in cases of fatal mammary cancer. Dr. Blundell says that he has never seen a coincident deposition in the breast and womb. The same thing may be said respecting the penis and prostate gland. Again, as bearing upon this point—the fallacy of the doctrine of sympathetic consentaneousness, in reference at least to the development of organic disease—it deserves to be noticed, that the organs most frequently affected with secondary cancer are the Liver and the Lungs; which cannot be said to have any very immediate sympathy with those parts, in which primary cancer is most frequently developed. That the veins are the real channels by which the contamination and transmission are effected, is very ably reasoned by our author upon the following grounds:—

"Cancerous matter exists in a multitude of cases in the veins of the diseased part; now this is obviously a most favourable circumstance for its circulation with the returning blood. The rapidity of the successive development of the disease in different organs, sometimes observed, seems only producible by the agency of a fluid which, like the blood, pervades them all. The liver and lung, the two organs in which foreign bodies introduced into the circulation are almost invariably observed to stagnate, are by far the most frequent seat of the secondary development of carcinoma. The parenchymatous viscera and the bones, the precise structures most frequently affected with secondary abscess, are those peculiarly liable to secondary cancer. In respect of both morbid products, the liver and lungs stand at the head of the list for frequency of implication. Secondary abscesses affect a special preference for the peripheric strata of the viscera; so likewise do secondary cancers. In the instance of the lung, I believe this readily explicable by the fact, that the majority of the ultimate ramifications of the pulmonary artery reach the periphery of the organ before becoming continuous with the capillaries, where stagnation must occur. Double organs are very rarely the simultaneous seat of primary abscess; in cases of secondary abscess both invariably suffer: the same propositions hold good of cancer. Secondary cancer in the liver and lung occupies the same elementary seat as the pus of secondary abscesses, a fact particularly insisted on by Cruveilhier. The lobules are the obvious seat of both products; and hence arises the similarity of form and outline exhibited by them both in the very early stages of formation. Add to all this the consideration that the generation of secondary, as a consequence of primary cancer, is in many cases otherwise utterly inexplicable; and a mass of argument is obtained, to which little solid objection can be offered." P. 107.

Encephaloid seems to be more liable to induce secondary carcinoma than even scirrhus: colloid ranks very low in this respect.—In the majority of cases, the secondary disease, whatever may have been the nature of the original growth, is of the encephaloid species;—scirrhus, however, is far from being uncommon.—Whenever carcinoma exists in several parts or organs at the same time, it is very generally of encephaloid kind. In a few cases, indeed, Scirrhus invades numerous organs; but commonly it is limited to one. In a remarkable case recorded by Velpeau, the number of organs and tissues involved in the cancerous disease was truly remarkable; viz. the cellular membrane, the muscles and bones, the lungs and heart, the tissue between the costal pleura and ribs, the stomach, duodenum and small intestines, the pancreas, kidneys, liver, vena cava, coats of the gall-bladder, the peritoneum, dura mater, and the thyroid gland were all, in various degrees, affected with the disease.

"The reproduction of the disease," says our author, "in its original locality in cases where, be it supposed, every single particle of morbid substance has been removed, can only be understood on the hypothesis that (the diathesis still remaining) the tissues originally affected retain their special attraction for cancerous germina." Query. Does this at all explain the matter? we fancy not. And here we may appropriately enquire, whether the examination of the blood and other fluids of the body has thrown any light upon the pathological history of Carcinoma. Dr. Walshe says that, in its advanced stages, he has occasionally remarked "a peculiar clamminess or stickiness" of the blood. Andral affirms that, he has in some instances detected pus-corpuscles in this fluid. But this can only be of occasional occurrence; as, even at advanced periods of the disease, when softening had been fairly established for some time, the microscope has utterly failed to detect the slightest difference in the physical characters of the blood. That, under such circumstances, there is often an excess in the proportion of the fibrine, and a decrease in that of the red globules, appears to be the result of the French pathologist's observation. Beyond this bare fact, hæmatological observations have not yet served to add any thing to the history of Cancer. As we have already alluded to the frequent discovery of carcinomatous matter in the veins—not only in the neighbourhood of, but at a distance from, the seat of cancerous formations—it is not necessary to say more upon this point.

COMPARATIVE PRONENESS OF DIFFERENT ORGANS TO CARCINOMA.

The organs, that are most frequently the seat of this morbid formation, are the Uterus, Stomach, female Mamma, Liver, Rectum, and Testicle.

No satisfactory explanation has yet been given of the superior liability in these parts. We shall, therefore, not dwell upon the subject; but pass on to notice one or two other points that call for attention, and can be more easily investigated.

That *sex* has something to do with the development of cancer is proved by the overwhelming excess of cases that occur in the uterus and female breast. The urinary organs in the male, we may remark, are, on the contrary, much more liable to be affected than the same parts in the female. *Age* also has clearly a marked influence in the production and localization of this disease. The general cellular membrane, the eye, the brain, and the lymphatic system, are the parts most commonly affected before the period of puberty; while the uterus, mamma, stomach, liver and intestines are seldom involved in the morbid process before the thirtieth year of life.—*Particular parts of organs* are more frequently attacked than others. On this point, Dr. Walshe says:

"Cancer of the œsophagus is excessively rare in the middle portion of the tube, most common in its upper third: the pyloric end is more commonly affected than the other regions of the stomach: the middle lobes of the brain suffer somewhat more frequently than the anterior and posterior; cancer of the uterus almost invariably originates in the cervix; the glans and prepuce are the chosen seat of cancerous formation in the penis; the cortical substance in the kidney; the cervix in the bladder.

"It is a curious fact, that, when one only of double organs is affected, the right suffers more frequently than the left. I have found the right lung to be nearly three times as often the seat of primary cancer as the left; and the left kidney not half so frequently affected as its fellow." P. 96.

Double organs are rarely found to be both affected with *primary* cancer, and are probably never simultaneously affected; whereas the *secondary* disease almost invariably implicates both, and generally originates in the two at one and the same time.

There is another fact, connected with this point, that may be noticed here. Secondary cancer is most frequently found in the peripheric portions of the viscera; the primary generally occupies the deeper parts of their substance.—The *different species of cancer* affect certain tissues and localities more than others. Encephaloid (although it has been found in every tissue and organ of the body) is more frequent than either scirrhus or colloid in the testicle, lungs, kidneys, spleen, and cerebral meninges; Scirrhus predominates in the uterus, the female breast, stomach, lower lip, and skin; Scirrho-encephaloid is, "par excellence," the hepatic cancer; while Colloid specially attacks the alimentary canal and omentum. We can suggest no rational interpretation of these local predilections.

*Symptomatology.*—We have not much to say upon this head, as every hand-book and treatise upon surgery contain sufficiently accurate descriptions of the usual local symptoms of cancer. There are only one or two points in the history of its progress that seem to call for a passing notice. The first of these is, as has been already remarked, that a cancerous growth often feels very much firmer and harder *in situ*, than after excision. Tumours of the mamma, that seemed to be almost as hard as marble while still attached, may feel flaccid and yielding when removed: "the absence of blood and of vital turgescence explains the change: the earliest alteration, produced in them by well-directed compression, is of the same kind." This remark applies more especially to Scirrhus; but even an Encephaloid growth, when confined within a tense cyst like that of the *tunica albuginea testis*, may give rise to the same deceptive phenomenon. In a diagnostic point of view, this circumstance is of high importance. Again, the presence or absence of pain must not be allowed to influence the opinion of the surgeon very much in his diagnosis. Pain seldom exists, at least severely, in the early stage of the morbid formation; nor is it even very uncommon for the disease to originate, advance, and terminate in the death of the patient, without being attended by very great suffering. Colloid is the species of cancer which most frequently exhibits this peculiarity. Great importance has been too often attached by surgical writers to the kind or character of the pain; it is generally described as being uniformly burning or lancinating. But this is a very uncertain condition: for it may not be present in actual cancerous growths; and, on the other hand, it may attend the development of such as are not at all malignant in their nature.

After mentioning the various lesions and morbid changes that are apt to occur in the soft solids during the progress of cancerous disease, our author thus describes the alterations that may take place in the *osseous system.*

"The following forms of morbid condition may be enumerated: 1. Atrophy of the spongy and compact structures from defective nutrition; 2. Excess of saline matter in their composition; 3. Insufficient supply of saline materials, in consequence of which, the bony tissue appears as if it had been macerated in a mineral acid (carnification); 4. Carcinoma of their substance. The first of these forms is singularly rare; Recamier, however, appears to have met with an example of it. Of the third, I have neither seen an instance, nor have met with a perfectly authentic description, and insert it rather hesitatingly. In the first three cases, the morbid state is a manifestation of the *cachexia;* in the fourth, an evidence of the *diathesis* having reached the bones. In the first, second, and fourth cases, especially the last, fractures occur with extraordinary facility. In cases of cancerous deposition in the bones, the fracture may either arise from complete transformation of a portion of the cylinder into cancerous matter, or from the pressure of a central growth, causing the absorption of the compact tissue. When the fracture is of the former description, crepitation will either be wholly absent or ill-marked: in the latter case this sign is producible in the ordinary way. When a cancerous patient suffers, more or less constantly, from pain in a particular spot in the course of a bone, the occurrence of such fracture is to be apprehended; but local pain is not a constant fore-runner of the accident, nor necessarily followed by it. The pain has been mistaken by patients for rheumatism; and this error has not unfrequently been shared by the medical attendant. In not a few cases the occurrence of fracture has manifestly hastened the fatal issue of the disease." P. 126.

*Progress and Terminations.*—Without doing more than simply enumerating the various ways in which a spontaneous cure of Cancer has been, it is affirmed, effected—(how rarely, we need scarcely say),—viz. resolution and absorption; conversion of the morbid substance into ossiform matter; suppuration; mortification; and cicatrisation after destruction by ulceration;—we wish to draw the reader's attention to the following interesting and important remarks on the *suspension* or temporary arrest in the progress of a cancerous formation that sometimes takes place. This circumstance is the more necessary to be generally known, from the erroneous but too common notion prevailing among medical men that serious organic disease cannot exist, when the symptoms very materially subside, or even entirely disappear, from time to time; in other words, when they exhibit any appearance of *intermittence*.

"No fact is better ascertained in the history of phthisis than that, in a certain proportion of cases (as yet undetermined), and under circumstances equally unknown, the disease undergoes suspension of progress, of greater or less duration. This change in the usual course of events may take place in any stage, the most advanced as the earliest, of pulmonary consumption. Now in such cases although the affection is, strictly speaking, anything but cured, yet a change is temporarily effected, which, as far as regards symptoms and the patient's feelings, amounts to delivery from his malady. A suspension of this kind occurs also, but more rarely, in cases of cancer; not a few instances are on record where women, affected with scirrhus of the breast in an active state, and even in the ulcerated stage, having refused to submit to the operation proposed to them, the morbid growth has subsequently fallen into a quiescent condition, and the patient's life not been shortened by her disease. It has occurred to me to become acquainted with two cases in which the patients succeeded in concealing for years the existence of cancer (in both instances ulcerated) from their husbands: the husband of one of these persons was a medical practitioner, a sufficient motive for presuming that neither the local suffering nor general disturbance were severe or obvious." P. 137.

The fatal issue of cancerous disease may arise either from the general depravation or cachexia that is ultimately induced, or from the exhaustion occasioned by continued pain and great discharge, or from the local disease obstructing and interfering with the performance of certain functions necessary to life, or from the supervention of some intercurrent disease, generally of an inflammatory nature, or lastly from hæmorrhage.

*Duration of the Disease.*—The data, which have hitherto been collected, are insufficient for any exact or very accurate conclusions upon this point. Cases have been known where a cancerous growth has existed for 30 or 40 years, and where the death of the individual seemed to be but little, if at all, accelerated by its existence. On the other hand, the disease has been known to prove fatal within two months from its primary development;—at least so far as *this* could be ascertained from the existence of symptoms. Perhaps we might fix a period of between three and four years as about the average duration of the disease, when not interfered with by any surgical operation. As a matter of course, the *species* of the cancerous growth has a very decided influence on the duration of the disease. Encephaloid is almost always much more rapid in its progress than true scirrhus: Colloid seems to be intermediate in this respect; it is neither so protracted as the very slow or chronic cases of Scirrhus, nor does it ever run its course so quickly as the more rapid or acute ones.

*Frequency and Mortality.*—Judging from our own Registration tables during the last five years, it would seem that a mean number of 2644 persons is annually cut off by carcinoma in England and Wales, or as nearly as possible 176 of every million alive in the country; and that 8045 per million of all deaths are owing to this disease. "If we suppose the disease to be in all instances necessarily fatal, and of the mean duration of three years and a quarter, it will follow that the number of persons, suffering from cancer in England and Wales at any given moment, averages 8595."

It has been supposed by M. Tanchou and others that the frequency of Carcinoma had been decidedly on the increase in Paris between the years 1830 and 1840; and M. Rigoni Stern has recently attempted to shew that, for the last 80 years, there has been a marked and steady increase in the disease (especially of the uterus) at Verona; but the calculations of these gentlemen are far from being at all satisfactory, much less are they conclusive, upon this point.

*Etiology.*—In spite of many assertions, in olden times at least, to the contrary, there is no satisfactory instance on record of carcinoma having ever been transmitted from one individual to another by infection, contagion, the direct inoculation of cancerous virus, or even the injection of this virus into the veins (of an animal). The occurrence of any malignant ulceration of the penis in men, who have long had intercourse with women labouring under cancer of the cervix uteri, is very rare indeed; so much so, that it is very questionable whether we can ever justly regard the two affections as standing to each other in the relation of cause and effect.

That the offspring of those, who have laboured under Cancer, are more liable than others to the development of the disease is a position, which, if not absolutely proved by the data which we at present possess, is at least highly probable. The family of Napoleon exemplifies the fact; himself, his father, and his sister Caroline, fell victims to cancer of the stomach. The period of life, at which Cancer proves most frequently fatal, is that between 35 and 70 years of age. Of 3036 cases, set down in the tables of the Registrar-General, 2395 appear to have occurred during that interval. Of the same total number, 199 occurred between 20 and 30 years, and 253 between 70 and 80 years, of age. Of the 3036 cases, 728 occurred in the male, and 2308 in the female, sex.* Hitherto it has been very generally asserted and believed that the tendency to cancer is greatest between the 35th and the 50th year of life. It would seem, however, from the more accurate researches of late years, that the mortality goes on steadily increasing until the 70th year, if not somewhat later.† The *mean* age, at the period of death, in 1200 cases of cancer was found by Dr. Walshe to be 57·2 years; in the male sex 59·4, and in the female 56·1 years.

Passing over the (alleged) influence of a variety of exciting causes—the unsatisfactoriness of the data, on which the opinions have been based, being but too obvious—we come to the following remarks on the supposed effects of modes and habits of life in the production of the disease.

" The foregoing survey, imperfect as it necessarily is, suffices to show that certain regions of the globe are peculiarly exempt from the ravages of cancer. But is this exemption to be really referred to the special influence of climate, or to some concomitant condition? Wherever the disease is particularly rare, it may be remarked that a low state of civilization prevails; wherever social organization is of a highly perfect kind, there cancer flourishes. May we, then, infer that, as has more than once been contended, cancer, like insanity, follows in the wake of civilization: and that as the ferment of a state of high social advancement is among the most active causes of destruction of intellect, so too it plays a prominent part in generating one of the most terrible physical evils to which humanity is subject? To answer this question satisfactorily would require

---

* The Paris registers, according to M. Tanchou, give 6967 deaths among females, and 2161 among males.

† The following table of 9118 deaths from cancer, occurring in Paris and its environs from the year 1830 to 1840, confirms the assertion here made.

| Ages. | Males. | Females. | Totals. |
|---|---|---|---|
| 1 to 10 | 9 | 14 | 23 |
| 10 — 20 | 13 | 13 | 26 |
| 20 — 30 | 62 | 169 | 231 |
| 30 — 40 | 190 | 822 | 1012 |
| 40 — 50 | 339 | 1636 | 1975 |
| 50 — 60 | 488 | 1620 | 2108 |
| 60 — 70 | 598 | 1469 | 2067 |
| 70 — 80 | 398 | 917 | 1315 |
| 80 — 90 | 62 | 273 | 335 |
| 90—100 | 4 | 22 | 26 |
| Totals . . | 2163 | 6955 | 9118 |

documents which are at present not to be had;—but the bias of evidence is most certainly in favour of the affirmative. And it is curious that even the lower animals appear to acknowledge a somewhat analogous influence; it will presently be seen that they are much more subject to the disease when in a state of domestication than in their natural wild condition." P. 161.

As far as we can judge from the researches which have been hitherto made, it would seem that town-life is not more favourable to the development of cancerous disease than country-life; in spite of the counter-assertion of M. Breschet. Nay, it has been asserted that the ratio of the mortality from this cause in the country is actually higher than that in cities and large towns. Grief and other depressing mental emotions have very generally been regarded as predisposing causes of cancerous disease.—Cancer is far from being uncommon in many of the lower animals: we have already alluded to this point, in our review of Heusinger's work on Comparative Pathology.

*Diagnosis.*—Under this head we shall briefly notice the other adventitious growths and structures, with which Carcinoma—including encephaloid, scirrhus, and colloid—has been often associated and confounded. It must be remembered that the genuine character or peculiarity of Carcinoma is its property of infiltrating the tissues in which it is developed, and eventually of contaminating the entire system: not so with the following formations.

1. Compound Cystoid Tumours, such as are so often seen in the ovary in cases of (what is erroneously called) encysted dropsy of this organ, have been deemed and termed malignant; yet there cannot be a reasonable doubt, says our author, but that they are quite unconnected with cancer, notwithstanding the assertion to the contrary of M. Cruveilhier in France, and of Dr. Hodgkin in this country. That a deposition of encephaloid or scirrhous matter may take place in the cysts of the diseased ovary, may be admitted (as we have previously remarked), without recognising any primary or essential malignancy in the ovarian disease. Dr. Walshe, after pointing out several inconsistencies in the views of the French pathologist, summarily disposes of those of the English one in the following terms:

"Dr. Hodgkin on his part, assuming that the compound ovarian cyst is the type of cancerous structure, is obliged to include the two forms of disease in the same anatomical category; he considers that there is in fact 'no appreciable difference either in the structure and arrangement of the cysts of gum cancer and encysted ovary, or in the composition of their contents.' (Op. cit. p. 291.) Nevertheless he formally denies the 'malignant' character of the compound ovarian cyst. Now he elsewhere (e. g. p. 268) distinctly makes the presence of the cystoid arrangement a test of 'malignity,' but, as the disease of the ovary in question possesses this arrangement in the most palpable manner, it follows that it is 'malignant:' hence the same formation is at once 'malignant' and 'non-malignant.' For my own part, I have not the slightest difficulty in rejecting compound cysts from the genus Cancer: where pathology is made the co-groundwork of classification with anatomy, this could not be otherwise." P. 179.

2. The Fibrous Growths or Tumours, which are not unfrequently developed in the substance or under the serous or mucous surfaces of the uterus, have been very differently viewed by different pathologists. By many, and these too of the highest authority, they have been regarded as utterly unconnected with cancerous development, and incapable of taking on the peculiar characters of carcinoma. Dupuytren, on the other hand, maintained that they frequently undergo true cancerous degeneration; while Dr. Hodgkin, and, following him, Dr. Ashwell view them as strictly and truly "scirrhous malignant growths," "genuine Cancerous productions." On this disputed question of practical pathology, our author, while unwilling to deny that fibrous tumours *may* ever become truly cancerous, distinctly states that he has never met with an instance of the change: the case, therefore, is one of extreme rarity, if indeed it is ever observed. That Dupuytren mistook certain fungating and intractable sores of the uterus for genuine cancerous ulceration, is now generally admitted. The following tabular view has been drawn up by our author to exhibit the prominent distinctive characters between Fibrous and Scirrhous tumours, and to shew the fallacy of the views entertained by Drs. Ashwell and Hodgkin.

| "FIBROUS TUMOURS. | SCIRRHOUS TUMOURS. |
|---|---|
| Frequently attain enormous bulk. | Rarely, if ever, while simple and in the purely tuberous form, exceed an orange in size. |
| Naturally assume a globular shape, and are of even outline. | Have a tendency to flattened shape, and more or less uneven outline, when they have attained any size. |
| Frequently occur in some numbers in the same organ; rarely in several organs of the same individual. | Are commonly solitary. |
| A curvilinear and concentric arrangement predominates in the fibrous stroma. | The fibriform substance, which forms the stroma of scirrhus, is frequently arranged almost rectilinearly. |
| Submitted to pressure, do not yield a thin whitish fluid. | Submitted to pressure, yield a thin whitish fluid. |
| Are comparatively opaque. | Thin slices especially have a more or less transparent aspect. |
| Their essential microscopical element is fibres, having the strongest similarity to those of natural fibrous tissue: cells are not to be descried. | Their essential microscopical elements are cells and granules. |
| Belong to the class of growths yielding gelatin. | Belong to the class of protein-growths. |
| Continuousness of their substance with surrounding structures is an accidental and rare condition. | The tendency to become continuous with and infiltrate surrounding tissues is one of their most strongly marked characters. |
| Frequently become the seat of calcareous ossiform-looking deposit. | I have never seen a specimen of true scirrhus, having ossiform-looking deposit in its interior (so-called 'ossification'), unless when the tumour sprang from some part of the skeleton. And even this is, in the case of scirrhous cancer, excessively rare. |

| FIBROUS TUMOURS. | SCIRRHUS TUMOURS. |
|---|---|
| Unless when producing serious local irritative action (itself a consequence commonly of their mechanical influence on surrounding parts), they do not affect the constitution. | Produce deep constitutional effects *per se*, and independently of all mechanical influence. |
| Unless under the last-mentioned circumstances, do not become the seat of softening. | Undergo softening as an attribute of their existence. |
| If partially removed, reproduction takes place, but of precisely the same substance as the original mass. | If partially removed, reproduction follows, but most commonly of a different species of cancer—the encephaloid. |
| If a fibrous tumour be removed completely, development of similar growths in distant parts does not ensue. | If totally and completely removed, development of cancerous products in other localities does, in a large proportion of cases, occur. |
| Unless when the seat of irritative action and softening, they do not excite irritation in the communicating lymphatic system: this irritation is never of a specific character. | Excite irritation in the lymphatic system, before any obvious local irritative symptoms have set in: this irritation is of specific charater, leading to development of substance in the glands of the same genus as the original growth. |
| Rarely, if ever (and certainly with less frequency than any of the natural vascularized tissues), present in their interior or on their periphery, small or large masses of scirrhous, encephaloid or colloid cancer. | Frequently present, either on their interior or at their periphery, small or large masses of the two other species of cancer; commonly of encephaloid more rarely of colloid." |

3. Erectile Tumors.—Cruveilhier has carried his peculiar notions respecting the seat of carcinoma in the veins to such an extravagant length that he actually asserts that "cancer is a varicose tissue, the meshes of which are filled with cancerous juice; and that varicose or venous erectile tisue is a cancerous structure, the meshes of which are filled with blood." That erectile structures (nævus maternus, aneurism by anastomosis, &c.), like compound cystoid tumours, are sometimes apt to become the seat of carcinomatous development, and consequently to give rise to all the constitutional symptoms of cancerous cachexy, cannot indeed be disputed; but these circumstances do not warrant the doctrine of the identity, or indeed of any close analogy between the morbid formations in question. In France, the term, "fungus hæmatodes," was long indiscriminately applied to fungating encephaloid, and to erectile vascular, tumours. This circumstance will in part account for the erroneous notions that still prevail among some of the continental writers. We may here mention that, some years ago, Dr. Hake of Brighton published a work to prove that carcinomatous and most other morbid growths essentially consist in a varicose state of the capillary vessels. Our readers will find an account of it in the No. of this Review for October 1839.

4. Enchondroma exhibits many of the outward features of Colloid.

"The spherical form, the uniform size, the thinness of the walls, and the semi-transparent aspect of the contents of the loculi in each, approximate these formations in point of external characters. But the following distinctions exist. On a section of fully developed colloid cancer the walls of the loculi appear sharply cut across at right angles with their plane; it is on the contrary extremely common to find the walls of the loculi of enchondroma exhibiting flat and extensive surfaces to the eye, as though the loculi had been opened to a small extent only. This probably depends upon a difference in amount of elasticity of the structure forming the walls in both growths. Colloid cancer does not originate in bone, which is the chosen seat of enchondroma. Enchondroma never infiltrates adjacent structures; colloid frequently does so affect them. Colloid never contains patches of bone, enchondroma does so almost invariably. Enchondroma yields chondrin, or in rare cases glutin; colloid furnishes no gelatin, and belongs to the protein-class of growths. Colloid affects the constitution *per se;* enchondroma simply (in the rare instances in which it ever does so) in the same manner as fibrous tumors." P. 182-3.

These observations may be compared with what has been already said under the head of Colloid.

5. Melanosis—under which term are included all accumulations of black colouring matter, whether fluid or solid, in normal or abnormal structures —has been regarded, by some of the leading pathologists of the day, as of a strictly cancerous nature. Dr. Walshe impugns the correctness of this opinion upon the following grounds:

"1. That the melanic pigment should in itself constitute cancer is an absurdity: it never even forms a stroma, as the cells continue permanently free. 2. The stroma of many melanic tumors is perfectly distinct in its physical, chemical, and microscopical characters from all cancerous stromata. 3. Many melanic tumors do not contain cancerous juice. 4. The microscopical characters of the pigment-cells and granules are the same in all kinds of growth in which they occur. 5. Melanic tumors, when no ordinary cancerous elements exist in them, cause no local or general symptoms, except those dependent on the size and seat of the growth. 6. When melanic tumors produce the local or general symptoms of cancer, they are found either to be composed of encephaloid or scirrhus, wholly or in part, impregnated with black pigment. 7. Neither the local nor general symptoms produced by carcinoma are modified in cases in which melanic matter is found to pervade it. 8. The circumstance that melanosis is rarely solitary, is strongly insisted on by Cruveilhier, as a ground for ranking it with cancer. But tubercle multiplies similarly, yet assuredly tubercle is not cancer." P. 184.

The opinion now generally entertained as to the cause of Melanosis is, that the pigmentary carbonaceous matter, which is naturally employed to colour different parts of the body, and more especially the hair, has become accumulated in the blood, and is gradually deposited in different parts and structures, abnormal as well as normal, of the body. The circumstance of melanotic deposits being much more frequent in grey and white horses than in those of a bay, brown or black colour, gives much probability to this opinion. Dr. Carswell's writings may be consulted with most advantage upon this pathological question.

6. Tubercle.—There is certainly no alliance, but, it would rather seem, an actual repulsion, between cancerous formations and those of a truly tuberculous character. The two rarely co-exist in the same individual; and moreover the organs, usually the seat of the one, are not those most frequently infested with the other. Of 104 cases of fatal cancerous disease, alluded to by Dr. Walshe, in seven only were the anatomical appearances of Phthisis discovered upon dissection. In five of these seven cases, the cancerous affection was Scirrhus; in the remaining two, it was Encephaloid, of which there were no fewer than 72 examples. This circumstance alone suffices to show the fallacy of the opinion of those writers, who have maintained that there is an alliance or connection between encephaloid and scrofulous disease. That cancerous formations and tuberculous deposits do occasionally exist together, Dr. W. does not deny; but he suggests that what has in many such cases been described as tuberculous matter, was really not so. The mere accumulation of fat in certain parts of an encephaloid growth will give these parts all the appearance of tuberculous deposition. In other cases, this phenomenon may occur in the way described in the following passage:

"A partial or general *yellowish tint* is sometimes produced in carcinoma, either, as in cases referred to by Laennec and Dr. Graves, by impregnation with the colouring matter of the bile; or, as is more usual, and, as has been just seen, by the changes undergone in the diseased mass in the vicinity of effused blood, resembling those observed in subcutaneous ecchymosis and cerebral hæmorrhage. Dr. Hodgkin expresses his belief, that the yellow tint occurs in the oldest parts of these growths; a remark which I have not been able to confirm. But there is much justness in the observation, that encephaloid thus discoloured bears some resemblance to tuberculous matter; a similarity which has caused the former to be sometimes mistaken for the latter." P. 82.

We must here draw to a close our observations on the pathological history of Cancer, and trust that we may have succeeded in presenting to the reader a fair and intelligible view of the more prominent and best ascertained phenomena of this terrible malady. What now remains is to notice briefly its *Treatment;* not, alas! that we have any thing new or very satisfactory to communicate upon this all-important subject; but in order to complete our general *aperçu* of the disease, and to point out the chief objects which the medical man ought to have in view in attempting to grapple with it. It has long been a matter of dispute whether carcinoma should be regarded *ab initio* as a local mischief eventually contaminating the whole system, or as a constitutional taint, manifested at first (it may be, for a length of time) by the formation of a morbid growth in one part of the body. The latter view is, as the reader will have been prepared to expect, that which is adopted by our author, who goes so far as to say that the following proposition—*a cancerous tumour under all circumstances, even should it remain single and stationary for years, is but the local evidence of a general vitiation of the system*—possesses "almost the certainty of mathematical demonstration." This language is strong; stronger, we suspect, than facts will warrant. The chief argument, be it remembered, adduced in its favour, is only one derived from the analogy of tuberculous deposits; "which" (although invariably proceeding from a constitutional cachexy and never from a merely local cause) "may exist for years at the summit of a lung, and every other organ may remain free to the last from the disease." But were we not taught by Dr. Walshe himself, at the very outset of his enquiry, to draw a line of marked discrimination between cell-matter that is altogether incapable of life and which is liable too to become converted into an amorphous and inorganic substance, and the development of those morbid cells which are possessed of a truly vegetative life, like those of Cancerous growths? Indeed, the whole of our author's remarks on the "Nature of Cancer" appear to us to be characterized much more by an ingenious spirit of speculation than by one of legitimate deduction from ascertained phenomena. For example, he takes for granted that "the blood and solids of the body are specially modified," *i. e.* altered from the healthy standard; although he admits, at the same time, that micro-chemical observations have hitherto completely failed in demonstrating any primary modification of these constituents of the living frame.—The exudation of the morbid blastemal fluid is supposed to take place "in consequence of local injury* or otherwise;" and, on this fluid, the system is presumed to have impressed an intrinsic power of vegetation.

"This vegetating faculty," continues Dr. W. "of the exudation re-acts on the system by constantly draining it of a portion of its nutrient materials,—the progeny feeds upon the parent organism, and the first phasis of evolution is accomplished. But the natural tissues have been so modified in properties by the constitutional state, that they are incapable of resisting the encroachments of the vegetating exudation, and hence become the seat of atrophous, ulcerative, and other modes of destruction. Discharges of various kinds now still further drain the system of its fluids, and impair its vital energies; and the second phasis is established. Meanwhile secondary alteration of the blood is effected; this fluid becomes the vehicle for the circulation through the system of elements possessed of a germinating force,—these stagnate, are deposited, and new local vegetations spring into life and activity. The same series of phenomena is again and again gone through; until the system, drained of its reparative fluids in feeding exudations and supplying discharges, exhausted of almost every drop of pure blood through the influence of secondary cancerous impregnation, paralysed in its nervous energies by physical anguish and deficiency of pabulum, sinks in the struggle against the superior powers of the new existencies it has created,—and in death is closed the third phasis of the disease." P. 190.

*Treatment.*—The Chapter upon this most important subject is very full and complete. Dr. Walshe has taken great pains to examine the alleged virtues of almost every remedy, and of every plan of medication that has been proposed. The details may perhaps be considered tedious and perplexing from their very multiplicity, and from the too frequent insufficiency of trustworthy data that have at different times been published; but, as the present Treatise has been evidently designed to furnish a complete history of Cancer, we must not find fault with our author. We shall rapidly follow him in his remarks. Under the head of *prophylactic treatment*,

---

* It may not be unworthy of notice, that the formation of hydatidic tumours is not unfrequently attributed by patients themselves to a blow or other injury upon the part. This circumstance will be found to be prominently alluded to by Klencke, whose recent work we reviewed in our last Number. We must confess that the idea—for it is nothing but a mere idea—keeps haunting our mind, that a sort of analogy may be traced between the development of hydatids, and that of cancerous tumors. Nor can we admit the conclusiveness of our author's objection to this hypothesis, "that no one has ever seen the imagined hydatids." May we not ask, with equal force, who has seen the blastemal fluid of cancer, or who has succeeded in detecting the modification of the blood that is supposed to exist?

Dr. W. lays it down as a rule that no mother, " whose relatives have suffered from cancerous diseases," ought to suckle her children. We greatly doubt the wisdom, and certainly do not believe in the necessity, of so wide-spread and sweeping an injunction as this ; more especially when it is considered that it is utterly out of the power of a woman, in the humbler classes, to procure " a vigorous nurse of healthy family" to suckle her offspring. If this practice of prohibiting suckling to women in whom there may be any hereditary tendency, however small or remote, to cancer, scrofula (as recommended by M. Lugol), and other constitutional maladies, were once to be generally adopted, how is a large portion of the infants of the poor to be reared at all? Medical men may be assured that hereditary maladies or diseased states of the system are not so very readily transmitted by the maternal milk, provided, all the while, the general health of the nurse be tolerably robust, and she do not commit the too common error of excessive or over-prolonged suckling. Much even may be done to correct, or compensate for, the faulty condition of a woman's milk by partly feeding the child, as well as by the judicious regulation of the mother's diet: but we cannot enlarge upon this subject at present.

Another hygienic suggestion of Dr. W. in reference to the children of those families in which there is believed to be any tendency to cancerous disease is, that the sons should not be educated to any profession or occupation involving a great deal of anxiety and care. Hence the bar, medicine, diplomacy, &c. should, he says, be avoided ; and " the speculations in which merchants, bankers, stock-brokers, &c. are so prone to indulge ought, if these occupations be embraced, to be systematically shunned." Females, our benevolent author adds, should not become governesses. It is all very well to say so ; but what are the poor creatures to do ; the life of a governess is surely better, and quite as free of disquietude and distress as that of a sempstress.

The following advice is of a more practicable character.

" Particular organs may have their special prophylaxis. The connexion of cancer of the penis with congenital phymosis is established with sufficient certainty to justify early circumcision in individuals, thus conformed, belonging to a cancerous family. Again, as certain morbid formations (*e. g.* erectile and compound cystoid growths) exhibit more than an average amount of liability to become cancerous, (I mean, through the development of scirrhus or encephaloid in their substance,) the removal of the former may be looked upon as prophylactic against the latter." P. 193.

The vast variety of *internal remedies*, that have been recommended at different times, for the cure of Cancer, is in itself sufficient evidence of the inefficacy of them all. If any one of them were possessed of real, positive, and substantial curative properties, we should not still be groping in the dark, as most assuredly we are. Let us cast our eyes over a few of them.

Conium has been immoderately praised by some writers, and unjustly undervalued by others. That its use, however prolonged, has ever effected a cure of genuine cancerous disease is more than doubtful; but that it may sometimes arrest its progress, as well as alleviate existing pain, cannot reasonably be denied. Recamier advises it to be taken in combination with the extract of sarsaparilla. Dr. Walshe has found it relieve pain and irritability, especially in cases of cancer of the stomach. Belladonna, Aconite, Stramonium, and other narcotics have been, at some time or another, recommended; but we possess no satisfactory evidence of any decided benefit, beyond the alleviation of pain, having ever been produced by their use.

Various preparations of Iron have been tried in the treatment of cancerous disease; and apparently with seeming advantage in some cases. Our author recommmends the iodide of this metal in preference to other preparations.

Mercury, even in minute doses, should generally be avoided: when carried to salivation, it is always injurious.—Iodine, externally and internally used, has unquestionably been found serviceable in a few cases: even a cure, it has been asserted by more than one respectable writer, has been effected by its employment. Dr. Walshe testifies to the truth of this statement from his own observation; and he adduces the recorded experience of Dr. Ashwell and Mr. Travers to the same effect. The remarks of the latter of these gentleman deserve particular notice. "By an indolent scirrhus I mean an incompressible permanent tumor, possessing for many years no distinguishing character of that disease; but in a deranged state of health assuming its genuine character, and at a particular period of life breaking up into an actual cancer. I do not entertain a doubt that such a tumor *may be, and often is, absorbed in its first stage, and need not therefore of necessity follow this course.* I feel assured that most of the hard breasts of young and middle-aged women admit of being reduced by the iodine or the mercurial ointment, if early resorted to, and steadily persevered in, *having often succeeded in procuring their absorption by such means, when, from the characters they presented, I should otherwise have felt compelled to urge their removal by operation. . . . . . Nothing is more certain than the susceptibility of absorption in many such tumors at an early stage of their existence.*" Dr. Walshe particularly enjoins that the ointment applied should not excite any irritation of the skin. He prefers the iodide of lead ointment (ʒj. to ℥j. of lard), as altogether unirritating, and as more discutient than any other. It should be smeared in very gently, for several minutes at a time, twice daily.

Arsenic unquestionably exerts, internally administered, a certain degree of salutary influence in some cases of scirrhous disease. Dr. Walshe appears to prefer the iodide—introduced into medicine by Dr. A. T. Thomson—in doses of from 1-16th to 1-12th of a grain twice daily, two hours after eating. The local symptoms often subside, and the general health improves. As to a real cure being ever effected, that is quite a different matter.

External Remedies.—Occasional application of *leeches*, in the neighbourhood of the affected part, is often of decided benefit; but "the obstistinate and senseless reiteration of leeching can only tend to weaken the patient, and sooner break up the constitution."—(Burns.) Of late years, several surgical writers have recommended the ligature of the main artery proceeding to a cancerous part, (the spermatic artery, for example, in cases of diseased testis); but the results of this practice do not encourage its repetition. We have already spoken of the effects of the external application of *Iodine*. The benefit to be sometimes derived in the treatment of cancerous tumours, from the methodic and regulated application of *compression*, was first clearly pointed out by the late Mr. Samuel Young. Since his time, the practice has been tried and approved of by many competent authorities. Mr. Travers has known tumours (such as those described in the extract previously given) "gradually reduced, and at length absorbed by equal and persevering compression, as by strips of soap and adhesive plaster, or, what is better, by an elastic roller passed many times round the chest, with layers of the Amadou smoothly interposed between the turns of the roller." M. Recamier has employed compression upon a very large scale, and the more important part of his results is as follows: "Of one hundred cancerous patients, sixteen appeared to be incurable, and underwent only a palliative treatment: thirty were completely cured by compression alone, and twenty-one derived considerable benefit from it: fifteen were radically cured by extirpation alone, or chiefly by extirpation and pressure combined, and six by compression and cauterisation: in the twelve remaining cases the disease resisted all the means employed." MM. Blizard and Masson have published three cases, and M. Carron du Villards three others,—in all of which irregularly nodular scirrhi, the seats of lancinating pain, &c., were removed by compression. Dr. A. L. J. Bayle (loc. cit.) gives, as the general results in 127 recorded cases, 71 cures, 26 instances of improvement, 30 of total failure. These results, the most favourable on the whole that can be adduced in favour of any mode of treatment, bear scrutiny of the severest kind. It is no doubt true, that, in some of the cases alleged to be cancerous, neither of the anatomical species of that affection existed; but it is on the other hand perfectly unquestionable that many of the absorbed growths were not only actually scirrhous, but had already become the seat of ulceration, when submitted to compression."

By far the best mode, in our author's opinion, of applying regulated compression, is by means of the ingenious apparatus contrived by Dr. Neil Arnott—to whom humanity at large was already so deeply indebted for more than one beautiful and most useful application of science to the relief of the suffering and afflicted. The inward satisfaction that he must experience in the consciousness of having been so great a benefactor of his race must be a much higher reward, to a man of his large and generous philanthropy, than all that wealth can procure or princes bestow.

But we must be on our guard against anticipating over-much from this, or indeed any other, outward application in the treatment of any such disease as Cancer. Would that the following flattering description held true in many cases!

"The effects produced by pressure are removal of existing adhesions, total cessation of pain, disappearance of swelling in the communicating lymphatic glands, gradual reduction of bulky masses to small, hard, flat patches or rounded nodules (which appear to be, both locally and generally, perfectly innocuous), and in the most favourable cases total removal of the morbid production. The relief of pain afforded by the instrument is, without exaggeration, almost marvellous; this effect is insured by the peculiar softness and other properties of the air-cushion, the medium through which the pressure of the spring is transmitted to the surface. Females unable to obtain sleep even from enormous doses of laudanum, cease instantaneously to suffer on its application; and sleep thenceforth, as though they were perfectly free from the disease." P. 211.

Dr. Walshe carries his admiration so far as to think that pressure on Dr. Arnott's system is applicable not only when the cancerous formation is situated over a bone, and where therefore a steady compression may be kept up, but even in other cases in which we should have deemed it quite inadmissible. "I see no reason," says he, "why cancer of the testicle might not be treated thus;—and gentle pressure on this plan deserves a trial in certain cases of cancer of internal parts (it would surely relieve pain), provided the general functional relations of those parts do not interfere (and they will often not do so) with the adoption of such pressure."

He has related the particulars of *one* case of a tumour of the mamma, presenting all the features of genuine scirrhus, in which a cure was effected by the use of the air-cushion compressor continued for between nine and ten months, the internal administration of the iodide of arsenic, and, (towards the close of the treatment), the external application of the iodide of lead ointment. Such is the treatment which, in our present state of knowledge, seems to promise the best chance of retarding, perhaps even of suspending, the progress of certain forms of carcinomatous disease; due attention being all the while paid, as a matter of course, to keep the nutritive and assimilative functions in a healthy condition, and to preserve the mind from anxiety and distress. Perhaps it would be judicious practice, in almost every case, to establish an artificial drain in some part by seton or issue; the effects of this remedy, in many chronic diseases, are occasionally of most unexpected advantage. Whether a medical man can rationally allow himself to entertain even a shadow of hope that a *specific* for cancer will ever be found, we shall not presume to determine. The reasoning of Dr. Walshe, on this point, is any thing but satisfactory to our mind; nor can we agree with him in his advice, whether as it respects Cancer or Phthisis—for it is in reference to these two diseases that his remarks apply—that "the efforts of those, who are placed in a position fitted for the purpose, should be unceasing in search of such a medicine; for nothing can be more unphilosophical than to conclude that it does not exist, because it has not yet been found."

Dr. Walshe canvasses at considerable length the important question as to the propriety of removing cancerous growths by surgical operation. This part of his work may be consulted by surgeons with great advantage. He demonstrates, with alas! too great success, the unsatisfactoriness of the results of excision, even under the most favourable circumstances; and he very nearly adopts the opinion enounced nearly 3000 years ago by the Father of Medicine, that even "occult" cancers should not be interfered with; experience having shewn that persons submitted to treatment perish more rapidly than those who have not been thus meddled with—'Οκόσοισι κρυπτοὶ καρκινοι γιγνονται, μὴ θεραπεύειν βέλτιον. θεραπευόμενοι γὰρ ἀπόλλυνται ταχέως. μὴ θεραπευόμενοι δὲ πλειω χρὸνον διατελοῦσι—Aphor. vi. 38. This sage remark might be applied, with no inconsiderable truth, to more diseases, we fear, than Cancer.

There is still some difference of opinion among medical men, as to the period or stage of the disease at which an operation may be undertaken with the best chances of success. Most surgeons recommend early extirpation, upon the ground that the longer that the primary local malady exists, the greater is the likelihood of lymphatie contamination, and of other evils supervening. It is, however, to be remembered, that the advocates of this practice invariably start from the notion of the absolute necessity of an operation in all cases ; and we have already seen reason to question the propriety of this opinion. That in many cases, where a scirrhous tumour might have remained stationary and dormant for a multitude of years if not interfered with, the disease has broke out with aggravated violence after the performance of an operation, cannot be doubted ; it is therefore quite unnecessary to adduce any illustrations of the fact.

"Again, 'it has been stated on high authority,' says Mr. J. Burns, Op. cit. t. i. p. 357,) 'that a woman has a better chance of a complete cure if the operation have been performed late than early, provided the disease continue limited, as there is a greater probability, in that case, that it remains local.' This seeming paradox (of which Galen may be regarded as the originator, for he taught that only fully formed cancers, and not those still occult, should be interfered with, with the cautery or knife,) has found defenders, indeed, in not a few practitioners; and recently MM. Hervez de Chegoin and Tanchou have warmly espoused it. According to the latter, cancer appears to localise itself with the progress of time under the influence of treatment, and loses proportionally its influence on the system; and so (as a matter of fact) cancer of the breast is known occasionally to undergo spontaneous suspension of progress, whereas M. Tanchou doubts the existence of a single case of real cancer having been cured by *early* operation. Some statistical results, published among the facts collected by M. Leroy d'Etiolles (loc. cit.), give unexpected support to the notion of the inutility of early, as contradistinguished to late, operation. Of 801 extirpations of cancers, 117 were performed less than a year after the appearance of the tumour; of these 117 operations 61 (or 52·1 per 100) were followed by reproduction within the course of the first year succeeding the use of the knife. And it is to be remarked that, at the time the author wrote, a full year had not elapsed since 112 of the 117 operations had been performed. On the other hand, among the operations not followed by reproduction there were 52 in which the morbid growth had, when removed, existed for five years. Finally, after careful examination of all the points and available documents bearing upon the question under discussion, I feel myself warranted in establishing the following proposition:—*Of a given number of cancerous individuals a considerably larger proportion will be saved from untimely death under the influence of well devised and judiciously sustained treatment, aided, if this become necessary, by extirpation performed at a comparatively late period, than will recover under the influence of the operation (unpreceded by methodised treatment) effected at the very earliest possible stage of local development.*" P. 239.

Such is the practical conclusion to which our author, after all his zealous investigation of the subject, has arrived. Many will differ from him, we believe; still, the opinion of one who has examined the history of the disease in all its bearings with so much attention, cannot but have considerable weight with the profession. We perhaps cannot do better than here recal to the mind of our readers the rules which Sir Benjamin Brodie has pointed out, for the guidance of the surgeon, in the extirpation of malignant tumours : they were published in the Lancet a year or two ago.

"When there is a general conversion of the glandular structure of the breast itself into a scirrhous structure, there being no well-defined margin, an operation never succeeds, but rather hastens the progress of the disease. When the skin is the seat of scirrhous tubercles, disseminated here and there around the tumour, or has become thickened and brawny, the pores being enlarged as if they were seen through a magnifying glass, there is no chance of making a permanent cure. When the nipple is retracted, the case is very unfavourable for an operation. In this case we may suspect that the disease has extended to the skin; and, if the skin in the neighbourhood of the nipple be examined, there will generally be found indications of disease in it. A dimple, or indentation over the tumour, is also an indication of the skin being affected, and renders the case unfavourable for operation. When the glands in the axilla are enlarged, not from simple irritation, but from cancerous affection, no ultimate cure can be expected. When the scirrhous tumour adheres to the pectoral muscle of the ribs, and when the skin is ulcerated, there is no chance of a permanent cure from operation. When there is evidence of disease of the liver, the lungs, the womb, or other internal organ, no permanent benefit can result from the removal of the breast. When all these cases are taken away, there are very few left in which it is right to operate."

What then are the cases in which amputation of a scirrhous mamma may be deemed proper? Sir Benjamin gives the following reply:

"When the skin is perfectly sound, when the nipple is not retracted, when there is no diseased gland in the axilla, when there is no sign of internal mischief, when there is no adhesion of the breast to the parts below, and when the patient is not very much advanced in life, I should say that there is a reasonable chance of an operation effecting a cure. I do not intend to say that, in all the excepted cases, there will be a permanent cure;—far from it; but there will be in some instances; and the chance of it may be sufficient to warrant you in recommending the patient to submit to the operation. I have the satisfaction of knowing that several persons, on whom I have operated under these circumstances, are now alive and well, but who would certainly (?) have been dead long since, had I not had recourse to it."

After the long and careful analysis which we have given of (the first half) Dr. Walshe's work, it only remains for us to return him our best thanks for the pleasure and instruction which we have received from its perusal. It is a volume of high and substantial merit, evincing on the part of the author an intimate acquaintance with all that has been written upon the subject of Cancer, and, at the same time, no ordinary acumen in sifting the opinions of others as well as in expounding and illustrating his own. Every medical man, who wishes to keep himself up to the existing standard of pathological science, must read it; and most who do this will, we feel assured, add it to their libraries, as an important work of reference.

